# Discrete mathematical network analysis bridging clinical vocabulary and patient discourse in interstitial cystitis/bladder pain syndrome online communications

**DOI:** 10.1038/s41598-025-30819-3

**Published:** 2025-11-29

**Authors:** Nobuo Okui, Kenta Ichino, Yuto Sakuma, Yoshihiro Ikehata, Machiko Okui, Shigeo Horie

**Affiliations:** 1 Data Science and Informatics for Genetic Disorders, Graduate School of Medicine, Juntendo University, Tokyo, 113-8421 Japan; 2 Urology, Yokosuka Urogynecology and Urology Clinic, Ootaki 2-6, Yokosuka, 238-0008 Kanagawa Japan; 3 Mathematics, Kanagawa Dental University, Inaoka-cyou 82, Yokosuka, 238-0008 Kanagawa Japan; 4 Urology, Graduate School of Medicine, Juntendo University, Tokyo, 113- 8421 Japan

**Keywords:** Diseases, Health care, Medical research, Signs and symptoms, Urology

## Abstract

Interstitial cystitis/bladder pain syndrome is a chronic condition involving pelvic pain and urinary symptoms. A three-stage analytical framework examined the correspondence between vocabulary from validated clinical questionnaires and language used in patient discussions on social media. Stage 1 identified 19 symptom-related terms from three questionnaires, all consistent with international diagnostic criteria. Stage 2 analyzed over 500,000 words from online discussions, detecting 73.7% of these terms and revealing a central “pain–urgency–voiding” triad in patient discourse. Stage 3 mapped strong symptom–site links, including burning–urethra and pain–abdomen, which may indicate underrecognized comorbidities. Clinical terms occupied central positions in the discourse network and showed greater structural importance than general vocabulary. Findings highlight differences between clinical terminology and patient-preferred language, suggesting strategies to improve assessment tools, address terminology gaps, and enhance patient-centered care. The approach is adaptable to other chronic conditions, supporting integration of real-world patient expression into clinical practice.

**Supplementary Information:**

The online version contains supplementary material available at 10.1038/s41598-025-30819-3.

## Introduction

Interstitial cystitis/bladder pain syndrome (IC/BPS) is a chronic condition characterized by bladder pain, pressure, or discomfort accompanied by lower urinary tract symptoms in the absence of identifiable pathology^1–6^.

Standardized symptom assessment is essential for consistent diagnosis and management.

Validated questionnaires including the Pelvic Pain and Urgency/Frequency (PUF) scale, Interstitial Cystitis Symptom Index (ICSI), and Interstitial Cystitis Problem Index (ICPI) have been widely adopted and endorsed by major urological associations^7–10^.

These instruments provide a structured framework for quantifying patient-reported symptoms, but may not fully capture the language patients use to describe their experiences in daily life.

Despite the central role of these questionnaires in clinical assessment, no previous studies have systematically examined the linguistic properties of their symptom terminology or validated this terminology against patient-generated language.

This gap limits our understanding of how well standardized clinical vocabularies reflect authentic patient experiences^11,12^.

Earlier studies have primarily focused on questionnaire validation and clinical utility, but none have employed linguistic or computational approaches to align clinical terminology with natural patient discourse^13,14^.

Meanwhile, large-scale digital health data and social-media communications have opened new opportunities to explore this relationship.

As artificial intelligence and natural language processing (NLP) become integral to healthcare, establishing a correspondence between validated clinical vocabularies and patient expressions is critical for improving data-driven symptom interpretation^15–19^.

Social-media platforms—particularly Reddit—provide unique access to spontaneous patient narratives, where individuals share experiences and symptom patterns in peer support environments^20–24^.

Such data offer valuable insights into how patients conceptualize and verbalize their symptoms, potentially revealing linguistic gaps in current assessment frameworks.

Network analysis provides a powerful computational approach for quantifying semantic relationships in patient discourse^25,26^. In our previous study, we introduced a discrete mathematical network analysis framework that applies graph-theoretical methods to characterize linguistic structures in social-media pain communication^14^.

By constructing co-occurrence networks of symptom-related terms, researchers can identify central concepts, thematic clusters, and the structural organization of patient narratives^14,27^.

This method allows large-scale quantitative exploration of patient communication, complementing traditional qualitative approaches and revealing relationships that may be overlooked in clinic-based assessments^11,12,28^.

Yet, to date, no studies have applied network analysis to IC/BPS or evaluated patient discourse in comparison with standardized symptom vocabularies.

Understanding these relationships is particularly important given the frequent coexistence of gynecologic comorbidities—such as vulvodynia, vestibulodynia, and pelvic-floor dysfunction—which complicate symptom interpretation and blur anatomical boundaries^4,29–38^.

Investigating how patients spontaneously describe these overlapping conditions may inform more nuanced and anatomically grounded clinical assessment.

This study aims to bridge validated clinical terminology with real-world patient discourse in IC/BPS through a comprehensive three-stage analysis.

In Stage 1, we systematically extracted and categorized symptom terminology from internationally validated questionnaires using rule-based linguistic methods^7,8,13,15–17^.

In Stage 2, we validated this clinical vocabulary against large-scale Reddit communications through network analysis, enabling quantitative mapping of linguistic relationships in patient-generated text^14,20–23,27,28^.

In Stage 3, we examined co-occurrence patterns between symptom expressions and anatomical sites to clarify how patients link pain descriptions with specific body regions^29,31–38^.

This reproducible computational framework provides an evidence-based bridge between standardized clinical assessment and patient-centered communication, contributing to both the refinement of symptom questionnaires and the advancement of NLP applications for chronic-pain research.

## Results

### Stage 1: clinical vocabulary extraction from questionnaires

Table 1 summarizes the distribution of 19 symptom-related terms identified by a rule-based linguistic analysis of three validated IC/BPS questionnaires (PUF, ICSI, ICPI), showing four distinct categories. Anatomical sites comprised the largest category (42.1%, *n* = 8), followed by pain-related and urinary-function symptoms (21.1% each, *n* = 4), with impact/bothering symptoms comprising the smallest category (15.8%, *n* = 3). This distribution illustrates the multifaceted nature of IC/BPS, affecting multiple anatomical locations with both sensory and functional domains.

**Table 1:** Complete symptom terminology extracted from IC/BPS questionnaires with source distribution.

Category	Terms	Count	Percentage	Source questionnaires
Pain-related symptoms	Burning, discomfort, pain, pressure	4	21.1%	PUF (pain), ICSI (burning, pain), ICPI (discomfort, pressure)
Urinary function symptoms	Frequent, need, urgency, urinate	4	21.1%	PUF (urgency), ICSI (frequent, need, urinate), ICPI (frequent, urinate)
Impact/bothering symptoms	Avoid, bother, symptoms	3	15.8%	PUF (avoid, bother, symptoms)
Anatomical sites	Abdomen, bladder, pelvis, perineum, sacrum, testes, urethra, vagina	8	42.1%	PUF (all 8 sites), ICSI (bladder), ICPI (bladder)
Total		19	100%	

PUF, Pelvic Pain and Urgency/Frequency Patient Symptom Scale; ICSI, Interstitial Cystitis Symptom Index; ICPI, Interstitial Cystitis Problem Index.

Questionnaire-specific analysis revealed distinct terminological emphases.

The PUF questionnaire demonstrated the most comprehensive vocabulary (15 of 19 terms), encompassing all anatomical sites and impact-related terminology, whereas ICSI and ICPI showed narrower scopes (6 and 5 terms, respectively) focused mainly on pain and urinary symptoms.

The automated extraction system achieved a precision of 94.7%, validating the accuracy of the rule-based approach.

Cross-referencing the extracted vocabulary with major IC/BPS diagnostic frameworks — including the American Urological Association (AUA) Guidelines, the European Society for the Study of Interstitial Cystitis (ESSIC) Classification, and the International Continence Society (ICS) Terminology — showed consistent alignment between questionnaire terminology and internationally recognized symptom criteria, including pain/pressure/discomfort and urinary urgency/frequency.

The overall alignment rate across these frameworks was 100%, showing that the extracted vocabulary accurately reflects the core domains of IC/BPS symptomatology.

### Stage 2: social media discourse analysis

Table 2 shows detection of the standardized 19-term clinical vocabulary in Reddit patient communications. Overall, 14 of 19 terms (73.7%) were detected among 5,202 unique terms forming 48,359 co-occurrence relationships. Detection rates varied across categories: pain-related symptoms showed complete detection (4/4 terms), with “pain” most frequent (*n* = 2,034), followed by “burning” (*n* = 71), “discomfort” (*n* = 3), and “pressure” (*n* = 1). Urinary-function symptoms showed partial detection (3/4 terms): “need” appeared with the second-highest overall frequency (*n* = 1,327), “urinate” was moderately frequent (*n* = 426), and “urgency” was rare (*n* = 7). The term “frequent” did not appear in patient communications.

**Table 2:** Clinical vocabulary detection in Reddit IC/BPS discourse.

Category	Detected terms	Frequency
Pain symptoms	pain (2,034), burning (71), discomfort (3), pressure (1)	2,109 total
Urinary symptoms	need (1,327), urinate (426), urgency (7)	1,760 total
Impact symptoms	avoid (281), symptoms (1,353)	1,634 total
Anatomical sites	bladder (2,217), abdomen (30), pelvis (40), urethra (24), vagina (49)	2,360 total
Overall	14 validated terms	7,863 total

Impact/bothering symptoms achieved 67% detection (2 / 3 terms), with “symptoms” showing high frequency (*n* = 1,353) and “avoid” at moderate frequency (*n* = 281), while “bother” was not detected. Anatomical sites demonstrated 63% detection (5 / 8 terms), led by “bladder” with the highest overall frequency (*n* = 2,217), followed by “vagina” (*n* = 49), “pelvis” (*n* = 40), “abdomen” (*n* = 30), and “urethra” (*n* = 24). The terms “perineum,” “sacrum,” and “testes” were not detected in patient communications.

**Fig. 1:**
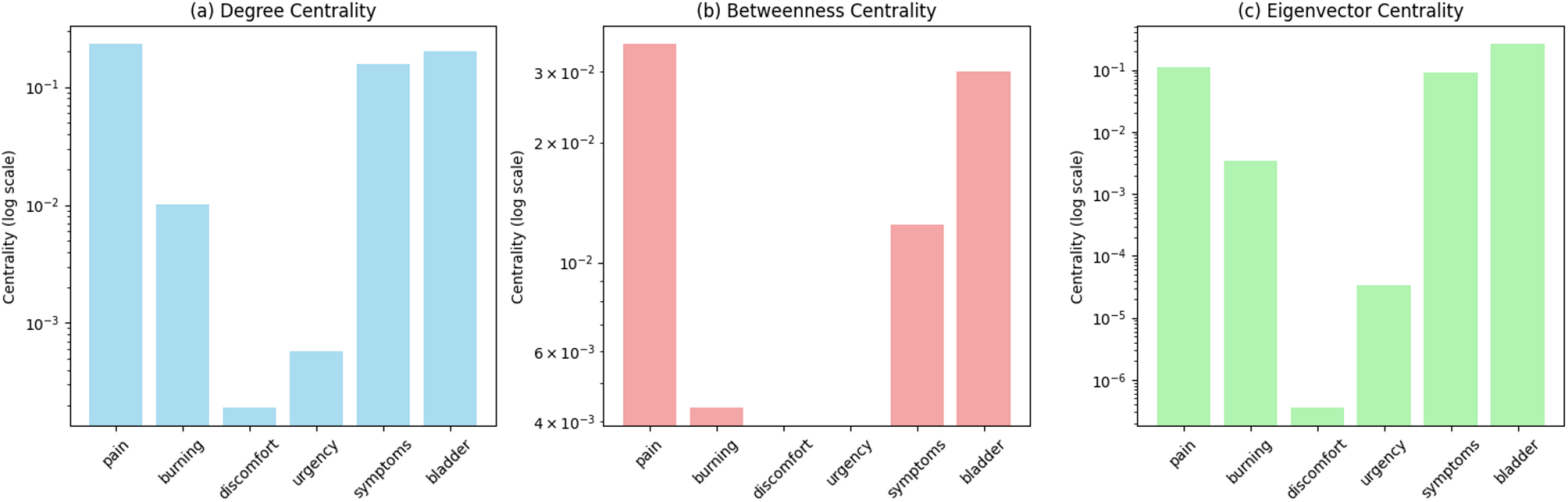
Centrality analysis of key IC/BPS terms in Reddit discourse. Bar charts of (**a**) degree centrality, (**b**) betweenness centrality, and (**c**) eigenvector centrality for six core clinical terms in patient communications. All measures on logarithmic scales (y-axis: centrality values, log scale; x-axis: clinical terms). Pain with consistently high centrality across all measures; bladder with particularly high eigenvector centrality, indicating connection to other highly connected terms.

Figure 1 illustrates the network centrality measures for key terms in the IC/BPS discourse network. “Pain” emerged as the primary semantic anchor with the highest degree centrality (0.2307), indicating direct connections to the broadest range of other terms. This central position illustrates pain’s role as the principal organizing concept in patient communications about IC/BPS. “Bladder” demonstrated the highest eigenvector centrality (0.2595), indicating connections to other highly connected terms and suggesting its role as a central anatomical reference point linking diverse symptom discussions. “Symptoms” showed high degree centrality (0.1569) and moderate eigenvector centrality (0.0923), positioning it as a general organizing term for symptom-related discourse.

Betweenness-centrality analysis identified terms acting as bridges between different semantic clusters. “Pain” again showed the highest betweenness centrality (0.0352), reinforcing its role in connecting diverse aspects of IC/BPS discourse. While “bladder” had high eigenvector centrality, it showed moderate betweenness centrality (0.0300), suggesting its role as a cluster center rather than an inter-cluster bridge. Validated clinical terms consistently outperformed general vocabulary across all centrality measures, showing higher average degree centrality (0.0589 vs. 0.0036, t = 17.30, *p* < 0.001) and betweenness centrality (0.0069 vs. 0.0004, t = 11.31, *p* < 0.001), indicating their structural importance within patient discourse networks.

**Fig. 2:**
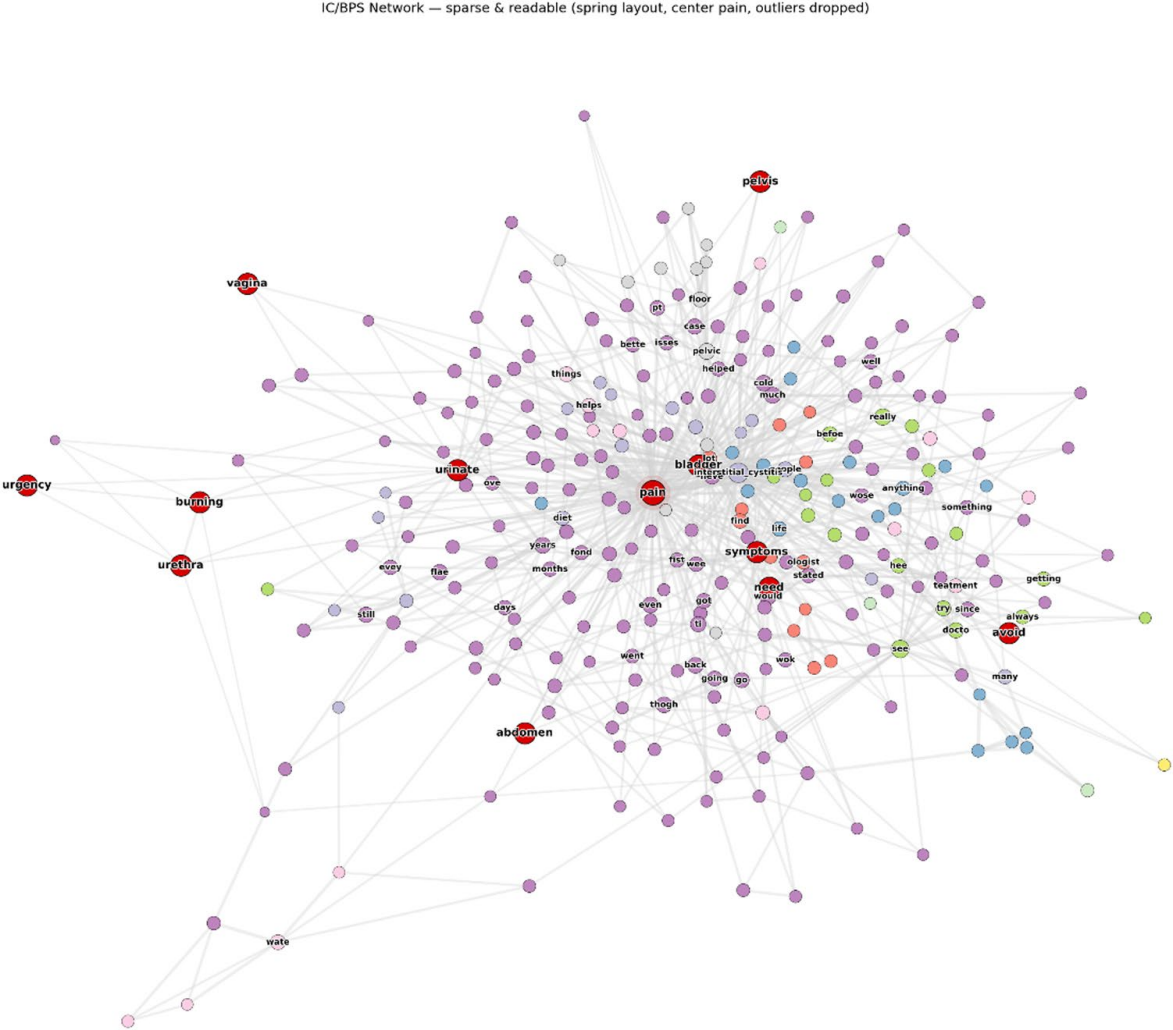
Network visualization of IC/BPS patient discourse. Spring-layout visualization of the co-occurrence network with pain at the center. Red nodes indicate validated clinical terms from standardized questionnaires; colored clusters represent thematic communities detected by the Louvain algorithm. Node size is proportional to degree centrality, and edge length inversely reflects co-occurrence strength (shorter edges = stronger connections). The network shows a clear hub-and-spoke organization around clinical concepts with distinct peripheral clusters. IC/BPS, interstitial cystitis/bladder pain syndrome.

Figure 2 illustrates the global structure of the IC/BPS discourse network.

The network was sparse overall (density = 0.0036) but exhibited a high clustering coefficient (0.4108), indicating strong local semantic coherence. It showed a scale-free degree distribution consistent with natural-language networks, where a few terms act as major hubs while most have limited connections. Connectivity analysis revealed multiple disconnected components, suggesting distinct thematic clusters in patient discourse. Despite the sparse global structure, the high clustering coefficient indicates formation of tightly knit semantic neighborhoods reflecting coherent thematic discussions.

Community detection using the Louvain algorithm identified 90 semantic clusters ranging from broad medical discussions to narrowly focused groups on specific symptoms or treatments. The largest community (2,227 nodes; 42.8%) represented general IC/BPS discourse integrating diverse symptoms and experiences. Secondary clusters showed thematic specialization: treatment and medication discussions (365 nodes; 7.0%), urinary symptoms and bladder function (358 nodes; 6.9%), pain management and relief strategies (322 nodes; 6.2%), and diagnostic or healthcare experiences (269 nodes; 5.2%).

Validated clinical terms appeared across multiple communities but were concentrated in the general discourse (64% of validated terms) and treatment-focused clusters (21%), suggesting that clinical vocabulary functions both as common organizing language and as specialized terminology within focused discussions.

Bootstrap analysis was performed to assess measurement stability across key network metrics. Resampling 80% of network edges across 100 iterations produced low variability in centrality measures for major terms. “Pain” showed a standard deviation of 0.0026 for degree centrality and 0.0027 for betweenness centrality, indicating highly stable measurements. “Bladder” demonstrated similar stability (degree SD = 0.0025; betweenness SD = 0.0027), confirming reliable identification of central terms. Less frequent terms exhibited greater variability, as expected: “urgency” showed degree-centrality SD = 0.0002, whereas “discomfort” displayed minimal variability owing to its extremely low frequency. Overall, the bootstrap results indicate that centrality estimates for the main clinical terms represent stable network properties rather than sampling artifacts.

The correspondence between detected patient vocabulary and established IC/BPS diagnostic frameworks was also examined. AUA Guidelines emphasizing bladder pain, pressure, and urinary urgency aligned closely with the highest-frequency detected terms (“pain,” “bladder,” “need”). ESSIC criteria focusing on chronic pelvic pain corresponded to “pain,” “pelvis,” and “bladder.” ICS terminology for storage symptoms and pain classifications showed strong correspondence with “urgency,” “urinate,” “pain,” “burning,” and “discomfort.” The overall alignment rate of 73.7% demonstrates substantial concordance between patient natural-language expressions and internationally recognized diagnostic terminology.

### Stage 3: Symptom–anatomical site co-occurrence analysis

Table 3 presents the strong co-occurrence pairs identified among the validated IC/BPS vocabulary terms using the strong-link criterion (distance = 1; edge-weight percentile ≥ 90%).

Nine prominent pairs were detected, with the highest weights observed for pain–symptoms (177; 97.2 percentile) and pain–urinate (163; 97.1 percentile), reflecting a pain-centric discourse structure coupled with voiding-related descriptions. Other high-ranking links included need–urinate (110; 96.4 percentile) and pain–need (54; 94.3 percentile), forming a tight pain–urgency–voiding triad that mirrors the clinical symptom complex of IC/BPS and suggests coordinated symptom experiences in patient discourse.

**Table 3:** Strong co-occurrence pairs (distance = 1, edge-weight percentile ≥ 90%) among validated IC/BPS vocabulary terms in Reddit discourse.

Rank	Term U	Term V	Category pair	Edge weight	Raw count	Percentile	U degree	V degree
1	Pain	Symptoms	pain_symptoms × impact_symptoms	177	173	97.2%	0.1964	0.1324
2	Pain	Urinate	pain_symptoms × urinary_symptoms	163	159	97.1%	0.1964	0.0580
3	Need	Urinate	urinary_symptoms × urinary_symptoms	110	106	96.4%	0.0505	0.0580
4	Pain	Need	pain_symptoms × urinary_symptoms	54	50	94.3%	0.1964	0.0505
5	Burning	Urethra	pain_symptoms × anatomical_sites	50	46	93.9%	0.0095	0.0031
6	Burning	Urinate	pain_symptoms × urinary_symptoms	37	33	92.0%	0.0095	0.0580
7	Burning	Bladder	pain_symptoms × anatomical_sites	37	33	92.0%	0.0095	0.0027
8	Pain	Abdomen	pain_symptoms × anatomical_sites	31	27	90.5%	0.1964	0.0041
9	Burning	Pain	pain_symptoms × pain_symptoms	30	26	90.2%	0.0095	0.1964

Edge Weight = enhanced weight including clinical bonus; Raw Count = co-occurrence count; Percentile = position among all network edges by weight; U/V Degree = degree centrality of each term.

Clinically significant anatomical-site associations emerged prominently for burning sensations, with burning–urethra (50; 93.9 percentile) representing the strongest anatomical link for burning symptoms, potentially reflecting urethral hypersensitivity commonly observed in IC/BPS patients. This association exceeded burning–bladder (37; 92.0 percentile) and was markedly stronger than burning–vagina (10; 60.4 percentile, below the strong-link threshold). Pain demonstrated broader anatomical associations, with pain–abdomen (31; 90.5 percentile) meeting the strong-link criterion, suggesting widespread pelvic involvement in patient experiences. The burning–pain self-reinforcing loop (30; 90.2 percentile) indicates that burning sensations and pain concepts are strongly interconnected in patient discourse, possibly reflecting shared nociceptive mechanisms.

The network architecture revealed hierarchical organization in which general symptom terms serve as primary hubs, whereas anatomical sites function as specialized nodes with targeted connections. Pain confirmed its role as the global semantic hub (degree centrality = 0.1964; z-score = 13.95; > 99.9 percentile), demonstrating exceptional connectivity across the entire discourse network. Anatomical nodes such as urethra (degree = 0.0031), bladder (0.0027), and abdomen (0.0041) showed lower global centrality but locally strong edges to specific symptom terms, indicating specialized anatomical-symptom clustering patterns. This structure suggests that patient discourse naturally organizes around symptom experiences first, with anatomical localization serving a secondary yet important specifying role in symptom characterization.

## Discussion

This study represents the first large-scale analysis of the relationship between systematically extracted symptom vocabulary from internationally validated IC/BPS clinical questionnaires and natural language patterns spontaneously used by patients in daily life and online communications^14–18,20–23^. In Stage 1, 19 clinical terms were extracted from three questionnaires (PUF, ICSI, ICPI) and shown to align fully with major international diagnostic criteria^1–3,9,10,13^. In Stage 2, 14 of these 19 terms (73.7%) were detected in Reddit patient discourse, indicating that a substantial proportion of clinical vocabulary is present in patients’ everyday language^20–23^. Network analysis further revealed that pain (degree centrality = 0.1964, z-score = 13.95, > 99.9 percentile) serves as the central organizing concept around which urgency and voiding behaviors are closely interconnected^14,23,27^. The symptom network architecture, termed the “pain–urgency–voiding triad,” reflects the recurrent co-occurrence of pain-related (“pain,” “burning”), urgency-related (“need”), and voiding-related (“urinate”) terms in patient discourse networks^14,23^. This structure emerged spontaneously in patient language and aligns with the established IC/BPS clinical syndrome, indicating that symptoms are experienced in an integrated manner^1–3,39^.

Stage 3 identified strong co-occurrence patterns between symptoms and anatomical sites, particularly burning–urethra (93.9 percentile) and pain–abdomen (90.5 percentile), revealing symptom associations concentrated in specific anatomical locations^14,23,27^. While burning–vagina did not meet the strong-link threshold, this finding suggests potential underreporting of vulvar symptoms or patients’ tendency to avoid expressing concerns about intimate areas^33,36–38^. These symptom–site patterns provide early indicators for gynecologic comorbidities including vulvodynia, vestibulodynia, and pelvic floor dysfunction^29,31–35,37,38^. Clinical practice should incorporate comprehensive evaluation encompassing vulvar, vaginal, and urethral assessments, particularly for patients reporting “burning” or “pressure” symptoms, in addition to bladder-focused evaluations^3,4,29,31–35,37–40^.

Our findings provide direct implications for next-generation IC/BPS questionnaire revision and development. Current standard questionnaires (PUF, ICSI, ICPI) rely primarily on frequency-based assessments^9,10,13^, but our results demonstrate the importance of symptom interconnections and anatomical localization^14,23,27^. New questionnaires should incorporate integrated symptom evaluation considering the pain–urgency–voiding triad^14,23^, anatomical mapping of burning sensations (particularly distinguishing urethra, bladder, and vulvar areas)^29,31–35,37,38^, and question items based on patient natural language patterns (such as “need to urinate,” “burning sensation,” “avoid activities”)^20–23^. Furthermore, standardized comorbidity screening is essential, requiring comprehensive approaches that simultaneously evaluate symptoms of vulvodynia and pelvic floor dysfunction^29,31–35,37,38^. Systematic incorporation of anatomical sites inadequately covered by existing questionnaires (vagina, perineum, sacrum)^29,31–34,40^ and diverse sensory expressions beyond burning and pressure could enhance assessment comprehensiveness and improve compatibility with patient self-expression while maintaining alignment with international standards^1–3^ and adapting to patients from different cultural and linguistic backgrounds^16,19^.

The 73.7% vocabulary detection rate provides an important benchmark for natural language processing system development^14–18,20–23^, while undetected terms (“frequent,” “bother,” “perineum,” “sacrum,” “testes”) offer crucial insights. Patients tend to prefer colloquial expressions over medical terminology (“need” instead of “frequent,” “avoid” instead of “bother”)^20–23^, emphasizing the necessity of integrating both clinical terms and patient language in patient-centered digital health system design^16,19–23^. The non-detection of male-specific terms (“testes”) may reflect gender bias in IC/BPS discourse on Reddit, raising concerns about insufficient representation of male patient voices^5,11,12,20–23^.

Network analysis revealed community structure comprising 90 semantic clusters, reflecting the diversity of IC/BPS patient experiences and supporting the need for individualized medical approaches^14,23,27,28^. The presence of treatment-related communities (7.0%), bladder function communities (6.9%), and pain management communities (6.2%) suggests that patients seek support focused on different aspects, providing a foundation for tailored treatment strategies^20–23,28^. These findings are applicable to precision medicine contexts for IC/BPS patient subtype classification^14,23,27,28^.

The hybrid approach developed in this study (rule-based linguistic analysis + clinical validation) presents a highly generalizable methodology applicable to other chronic diseases^14–18,27,28^. Similar vocabulary gaps likely exist in disease areas centered on subjective symptoms, such as irritable bowel syndrome, fibromyalgia, and chronic fatigue syndrome^11,12,23,28^. Future development of multi-disease symptom vocabulary databases and comparative analysis of language patterns across diseases becomes feasible^14–18,23,27,28^. Furthermore, expansion of technological foundations for real-time symptom tracking, treatment effect monitoring, and automation of patient-reported outcome measurements is anticipated^15–18,27,28^.

Study limitations include that Reddit users self-identified as having IC/BPS symptoms without formal diagnostic verification, and restricted generalizability due to demographic bias among Reddit users (young, tech-savvy, English-speaking, female-predominant)^20–23^, insufficient capture of dynamic symptom progression changes due to cross-sectional design^14,23,27^, and difficulty in complete separation of complex interactions among comorbid conditions^29,31–38^. The symptom overlap between IC/BPS and vulvodynia particularly demonstrates limitations of single-disease analysis^29,31–38^. Future research requires validation across multiple languages and cultures^16,19^, longitudinal symptom tracking^14,23,27^, and more comprehensive modeling considering comorbid conditions^29,31–38^.

This research demonstrated the effectiveness of computational linguistic methods in bridging clinical assessment tools with authentic patient experiences, providing an important foundation for developing patient-centered approaches in IC/BPS care. The network analytical framework in this study draws on established applications of graph theory and discrete mathematics in urological research, which have been successfully applied to treatment decision support, dynamic symptom clustering, and social media discourse analysis in chronic pain and urinary disorders^41–44^. Quantitative understanding of patient natural language patterns enables the development of next-generation digital health applications, individualized medical strategies, and truly patient-centered symptom evaluation tools, representing transformative insights for the field.

## Conclusions

This study established a reproducible three-stage computational framework linking validated clinical terminology with patient-generated discourse in IC/BPS. Nineteen symptom terms extracted from internationally validated questionnaires (PUF, ICSI, ICPI) were cross-validated with large-scale Reddit communications, detecting 73.7% concordance between clinical and natural language expressions. Network analysis identified a “pain–urgency–voiding” symptom triad and strong symptom–site associations (e.g., burning–urethra, pain–abdomen), while additional anatomical terms—such as vagina, perineum, and sacrum—were underrepresented in current assessment tools. These findings provide quantitative linguistic evidence for refining IC/BPS questionnaires, enhancing patient-centered evaluation, and informing natural language processing applications in digital health. This framework may be extended to other chronic pain disorders characterized by complex, language-dependent symptomatology.

## Methods

### Study design and data sources

This study followed a reproducible three-stage analytical framework designed to connect validated clinical terminology for interstitial cystitis/bladder pain syndrome (IC/BPS) with real-world patient discourse^3,14,27^.

Stage 1 extracted standardized symptom terms from validated IC/BPS questionnaires.

Stage 2 validated these terms against large-scale social-media communications.

Stage 3 analyzed co-occurrence patterns between symptoms and anatomical sites within patient discourse.

The analysis used three internationally validated questionnaires — the Pelvic Pain and Urgency/Frequency (PUF) scale (8 questions, score 0–35), the Interstitial Cystitis Symptom Index (ICSI; 4 questions, score 0–20), and the Interstitial Cystitis Problem Index (ICPI; 4 questions, score 0–16).

All questionnaires were obtained in their original English versions as published in peer-reviewed literature and clinical guidelines^2,9,10^.

Social-media data for Stages 2 and 3 were collected from Reddit, a discussion-based platform hosting active patient communities for chronic conditions.

Posts containing the term “interstitial cystitis” were retrieved between August 2024 and July 2025, yielding 245 original posts and 4,697 comments (total 525,465 words).

Reddit’s pseudonymous format promotes open discussion of intimate symptoms, providing an ethically appropriate data source for pain-related discourse.

All data were publicly available and analyzed in compliance with Reddit’s terms of service and established research-ethics guidelines^20,22,27^.

### Stage 1: clinical vocabulary extraction from questionnaires

This stage aimed to extract a standardized set of clinically validated symptom terms from established IC/BPS questionnaires, using a two-stage algorithmic filtering process designed for reproducibility and to eliminate subjective bias^6,15,17^.

#### Linguistic filtering

Automated part-of-speech analysis (NLTK averaged-perceptron tagger) identified candidate terms meeting inclusion criteria: nouns (NN, NNS) for pathological states, anatomical sites, or symptoms; adjectives and participles (JJ, VBG, VBN) for symptom characteristics; and verbs (VB) for symptom experiences or related behaviors. Tokens were restricted to alphabetic characters with a minimum length of three to exclude abbreviations and artifacts.

#### Exclusion filtering

Sixty-five categories of non-symptom terms were removed, including adverbs of degree (e.g., never, usually), quantitative expressions (numbers, temporal quantifiers), structural words (articles, prepositions), instruction vocabulary (question, score), and modifiers lacking symptom relevance (little, during, after).

This exclusion list was iteratively refined through pilot testing to optimize precision while maintaining recall^15,17,18^.

Compound expressions were automatically decomposed into single words (e.g., “burning pain” → “burning” + “pain”) to ensure consistent vocabulary capture and cross-study comparability.

The filtered output was then compared with a curated reference vocabulary of 19 validated IC/BPS symptom terms derived from international diagnostic frameworks^3,14,17^.

Each validated term was automatically assigned to one of four predefined categories—pain-related, urinary-function, impact/bothering, or anatomical-site symptoms—while novel terms were flagged for expert review.

#### Clinical alignment and diagnostic validation

Validation of the extracted clinical vocabulary was performed through automated cross-referencing with three internationally recognized diagnostic frameworks: AUA Guidelines (2011, 2014), ESSIC Classification, and ICS Terminology^1–3^.

Exact string matching and semantic-equivalence testing confirmed that all 19 extracted terms corresponded to the symptom domains defined in these standards, including pain / pressure / discomfort and urinary urgency / frequency.

This cross-validation ensured that the vocabulary derived from standardized questionnaires accurately represents the clinically accepted symptomatology of IC/BPS.

### Stage 2: social media discourse analysis pipeline

This stage validated the standardized clinical vocabulary against patient-generated language on Reddit to evaluate correspondence between formal assessment terminology and spontaneous patient communication^14,17,20^.

#### Text normalization

A customized normalization map corrected 47 misspelled medical terms (e.g., “intestitial” → “interstitial,” “theapy” → “therapy”) and translated 23 social-media colloquialisms (e.g., “pee” → “urinate,” “hurt” → “pain,” “ic” → “interstitial_cystitis”).

This process unified informal patient expressions with standardized clinical terminology while preserving semantic meaning.

#### Clinical-term prioritization

Building on Stage 1, the established 19-term validated vocabulary was used as a reference for automatic classification into four predefined categories—pain-related, urinary-function, impact/bothering, and anatomical-site terms.

Validated terms were retained regardless of frequency, ensuring that key symptom expressions were not lost during filtering.

#### Tokenization and filtering

Enhanced tokenization with part-of-speech analysis (NLTK averaged-perceptron tagger) extracted medically relevant tokens, removed stop words, and excluded items shorter than two characters^14,15,17^.

This pipeline enabled reproducible mapping of patient discourse to validated IC/BPS terminology.

#### Clinical alignment and diagnostic validation

To assess clinical correspondence between patient-generated language and standardized diagnostic terminology, detected symptom terms from Reddit discourse were compared with the AUA Guidelines, ESSIC Classification, and ICS Terminology.

The highest-frequency detected terms—such as “pain,” “bladder,” “need,” “urgency,” and “burning”—showed strong alignment with the core diagnostic features of IC/BPS, confirming that patient discourse largely reflects internationally recognized clinical symptom categories.

### Stage 3: Symptom-anatomical site co-occurrence analysis

This stage identified prominent associations between symptom expressions and anatomical localizations in patient discourse and quantified their co-occurrence patterns^14,17,27^.

The same Reddit dataset described in Stage 2 (245 original posts and 4,697 comments; 4,942 documents) was used for this analysis.

After preprocessing, these documents yielded 122,347 lexical items for co-occurrence computation.

#### Dataset and co-occurrence data Preparation

This stage aimed to identify prominent associations between symptom expressions and anatomical localizations within patient discourse and to quantify their co-occurrence patterns^14,17,27^.

A co-occurrence dataset was constructed from 122,347 Reddit posts and comments containing the term “interstitial cystitis” collected over a 12-month period.

Texts were lower-cased, spelling-corrected, and normalized to the validated 19-term IC/BPS clinical vocabulary encompassing four categories: pain-related, urinary-function, impact/bothering, and anatomical-site terms.

Non-clinical stop words were removed using a medical-domain exclusion list optimized for social-media discourse.

This preprocessing produced a clean lexical dataset suitable for downstream network construction.

#### Construction of the symptom–site co-occurrence network

Semantic relationships between terms were captured using a sliding-window approach (window = 5 tokens), in which co-occurrence frequencies were calculated for all term pairs appearing within the same contextual span.

Edges with fewer than five co-occurrences were discarded to remove noise while retaining clinically meaningful associations.

To preserve clinical salience, edges connecting validated clinical terms received an additional weight bonus (+ 2) beyond raw co-occurrence counts.

This weighting ensured that the standardized vocabulary maintained visibility within the overall network structure, even in the presence of frequent general language.

#### Quantification of network structure and symptom–site clusters

The resulting network was analyzed using graph-theoretical metrics to characterize structural relationships between symptoms and anatomical references.

Degree centrality identified highly connected symptom concepts, betweenness centrality revealed bridging terms connecting semantic clusters, and eigenvector centrality highlighted terms associated with other highly connected nodes^14,17,27^.

Eigenvector centrality was computed using iterative convergence criteria (max_iter = 2000; tolerance = 1 × 10⁻⁶), with fallback procedures for non-convergent cases.

Community detection employed the Louvain algorithm with clinical weighting to identify thematic clusters and optimize modularity.

All pairwise relationships among the 19 validated clinical terms were quantified by enhanced edge weight, raw co-occurrence count, percentile rank, shortest-path distance, Jaccard coefficient, and individual centrality scores.

#### Strong-link identification and validation

“Strong links” were defined a priori as pairs with shortest-path distance = 1 (direct co-occurrence) and enhanced edge-weight percentile ≥ 90% among all network edges^14,17,27^.

This stringent threshold identified only the most prominent symptom–anatomical associations in patient discourse.

Particular focus was given to co-occurrences connecting symptom terms (pain-related, urinary-function, and impact categories) with anatomical sites (bladder, urethra, vagina, pelvis, abdomen, perineum, sacrum, testes).

#### Bootstrap validation and stability assessment

Statistical validation compared network metrics for validated clinical terms versus general vocabulary^14,17,27^.

Two-sample t-tests evaluated differences in centrality measures (α = 0.05).

A bootstrap resampling procedure (100 iterations, 80% edge resampling per iteration) assessed measurement stability across key centrality metrics.

External criterion validation was performed by cross-referencing the identified strong links with the 19-term clinical vocabulary to verify semantic alignment between patient-generated expressions and standardized IC/BPS terminology.

Coverage and stability statistics were computed for each symptom category, confirming that the observed network properties reflect genuine structural patterns rather than sampling artifacts.

### Statistical analysis and computational framework

Statistical analysis employed automated descriptive statistics to characterize the distribution of extracted terms across predefined categories, with percentage distributions calculated algorithmically for each symptom category. The system computed precision metrics by calculating the ratio of validated medical terms to total extracted terms, recall metrics through comparison against the reference vocabulary, and F1-scores for overall performance assessment. Automated frequency counting and proportional analysis were applied to describe the relative representation of symptom domains within the extracted vocabulary, providing quantitative characterization of terminological emphasis across the three questionnaires.

A Python 3-based automated extraction system was implemented using the Natural Language Toolkit (NLTK version 3.8+) for systematic symptom terminology identification. Specific dependencies included punkt tokenizer, averaged_perceptron_tagger for part-of-speech analysis, and wordnet for semantic processing. The system employed robust text encoding detection supporting multiple character encodings (UTF-8, Latin-1, Shift_JIS), followed by automated text preprocessing including whitespace normalization and special character handling. Comprehensive error handling included graceful degradation for NLTK resource failures with fallback to regular expression-based tokenization and detailed logging of processing statistics.

Statistical significance was defined as *p* < 0.05 for all analyses. Normality of continuous variables was assessed using the Shapiro-Wilk test. For normally distributed data, two-sample t-tests compared centrality measures between clinical and general vocabulary terms. For non-normally distributed data, Mann-Whitney U tests were applied. Effect sizes were calculated using Cohen’s d for parametric tests and rank-biserial correlation for non-parametric tests. Multiple comparisons were adjusted using the Benjamini-Hochberg false discovery rate correction where applicable. Categorical variables were analyzed using chi-square tests or Fisher’s exact tests when expected cell counts were below 5. Network-specific metrics including clustering coefficients, path lengths, and modularity scores were computed using established graph theory algorithms. Confidence intervals were calculated at the 95% level for all point estimates.

All analysis code, preprocessing scripts, and network data files were implemented with full reproducibility specifications. The analysis pipeline supports multiple input formats and produces structured outputs including term lists, centrality rankings, community assignments, and statistical summaries in both human-readable and machine-parseable formats for downstream analysis and integration with larger research workflows. The computational environment consisted of Windows 11 (Microsoft Corporation, Redmond, WA, USA) as the operating system, Python 3 (Python Software Foundation, Wilmington, DE, USA) as the programming language, with programming assistance from ChatGPT-4 (OpenAI, San Francisco, CA, USA), code execution in Google Colab (Google LLC, Mountain View, CA, USA), and data collection from Reddit (Reddit Inc., San Francisco, CA, USA).

## Supplementary Information

Below is the link to the electronic supplementary material.

Supplementary Material 1

## Data Availability

The datasets generated and analyzed during the current study are available in the Zenodo repository, at [https://doi.org/10.5281/zenodo.17423755](https:/doi.org/10.5281/zenodo.17423755) . This dataset includes all derived network data (node lists, edge lists, centrality metrics, and category summaries) corresponding to the figures and tables presented in this manuscript. The original text data were obtained from the public discussion forum Reddit (www.reddit.com) and are not redistributed here in accordance with the platform’s terms of use.

## Code availability

All code used for preprocessing, co‑occurrence network construction, community detection, centrality analyses, and figure generation is openly available at:

https://github.com/Curiosity-Mars/NLP-Interstitial-Cystitis/. The repository includes Colab‑ready scripts and instructions to reproduce the results reported in this study. Any third‑party dependencies and exact version pins are listed in the repository. No proprietary software is required.

## References

[1] van de Merwe, J. P. et al. Diagnostic criteria, classification, and nomenclature for painful bladder syndrome/interstitial cystitis: an ESSIC proposal. *Eur. Urol.* **53**, 60–67 (2008). 17900797 10.1016/j.eururo.2007.09.019

[2] Hanno, P. M. et al. Diagnosis and treatment of interstitial cystitis/bladder pain syndrome: American urological association guideline (2011; amended 2014, 2022). *J. Urol.* **208**, 34–42 (2022). 35536143 10.1097/JU.0000000000002756

[3] Doggweiler, R. et al. A standard for terminology in chronic pelvic pain syndromes: A report from the chronic pelvic pain working group of the international continence society. *Neurourol. Urodyn.* **36**, 984–1008 (2017). 27564065 10.1002/nau.23072

[4] Sullivan, M. E. et al. Role of gynecologic findings in interstitial cystitis/bladder pain syndrome: A consensus. *Neurourol. Urodyn.*10.1002/nau.70099.( (2025). 40575937 10.1002/nau.70099 PMC12748013

[5] Berry, S. H. et al. Prevalence of symptoms of bladder pain syndrome/interstitial cystitis among adult females in the United States. *J. Urol.* **186**, 540–544 (2011). 21683389 10.1016/j.juro.2011.03.132 PMC3513327

[6] Clemens, J. Q. et al. Development, validation and testing of an epidemiological case definition for interstitial cystitis/painful bladder syndrome. *J. Urol.* **183**, 1848–1852 (2010). 20303099 10.1016/j.juro.2009.12.103 PMC3519367

[7] Uguzova, S. et al. Current status of patient-reported outcome measures and other subjective assessment grading tools in bladder pain syndrome. *Int. Urogynecol. J.* **34**, 1677–1687 (2023). 37129626 10.1007/s00192-023-05551-z

[8] Taneja, R. et al. Validation study of new clinical scoring – Apollo clinical scoring system for bladder pain syndrome/interstitial cystitis and comparison with standard O’Leary-Sant score. *Int. Urogynecol. J.* **35**, 1137–1144 (2024). 37642668 10.1007/s00192-023-05641-y

[9] Parsons, C. L. et al. Increased prevalence of interstitial cystitis: previously unrecognized urologic and gynecologic cases identified using a new symptom questionnaire and intravesical potassium sensitivity. *Urology* **49** (5A Suppl), 58–63 (1997). 12385909 10.1016/s0090-4295(02)01829-0

[10] O’Leary, M. P. et al. The interstitial cystitis symptom index and problem index. *Urology* **49** (5A Suppl), 58–63 (1997). 9146003 10.1016/s0090-4295(99)80333-1

[11] Nickel, J. C. et al. Patient and physician perspectives on interstitial cystitis/bladder pain syndrome: a gap in understanding. *Urology* **79**, 112–118 (2012).

[12] FitzGerald, M. P. et al. Patient perspectives on the impact of interstitial cystitis/bladder pain syndrome and unmet needs: results from a national survey. *BMC Urol.* **19**, 43 (2019).31146773

[13] Clemens, J. Q. et al. Validation of a modified National Institutes of Health chronic prostatitis symptom index to assess symptoms of interstitial cystitis/painful bladder syndrome. *Urology* **64**, 807–811 (2004).

[14] Okui, N. & Horie, S. Natural language processing reveals network structure of pain communication in social media using discrete mathematical analysis. *Sci. Rep.* **15**, 29219 (2025). 40783425 10.1038/s41598-025-14680-y PMC12335456

[15] Wang, Y. et al. Clinical information extraction applications: A literature review. *J. Biomed. Inf.* **77**, 34–49 (2018). 10.1016/j.jbi.2017.11.011 PMC5771858 29162496

[16] Spasic, I. & Nenadic, G. Clinical text data in machine learning: systematic review. *JMIR Med. Inf.* **8**, e17984 (2020). 10.2196/17984 PMC7157505 32229465

[17] Kersloot, M. G. et al. Natural language processing algorithms for mapping clinical text fragments onto ontology concepts: a systematic review and recommendations for future studies. *J. Biomed. Semant.* **11**, 14 (2020). 10.1186/s13326-020-00231-z PMC7670625 33198814

[18] Zhou, B., Yang, G., Shi, Z. & Ma, S. Natural language processing for smart healthcare. *IEEE Rev. Biomed. Eng.* **17**, 4–18 (2022). 10.1109/RBME.2022.3210270 36170385

[19] Himmelstein, G., Bates, D. & Zhou, L. Examination of stigmatizing language in the electronic health record. *JAMA Netw. Open.* **5**, e2144967 (2022). 35084481 10.1001/jamanetworkopen.2021.44967 PMC8796019

[20] De Choudhury, M. & De, S. Mental health discourse on reddit: self-disclosure, social support, and anonymity. *Proc. Int. AAAI Conf. Web Soc. Media*. **8**, 71–80 (2014).

[21] Park, A. & Conway, M. Longitudinal changes in psychological states in online health community members: Understanding the long-term effects of participating in an online depression community. *J. Med. Internet Res.* **19**, e71 (2017). 28320692 10.2196/jmir.6826 PMC5379019

[22] Adeyemi, T. et al. Reddit as a social media self-management tool for inflammatory bowel disease: qualitative analysis. *J. Med. Internet Res.* **27**, e75137 (2025). 40749205 10.2196/75137 PMC12316438

[23] Nunes, D. A. P., Ferreira-Gomes, J., Neto, F. & Martins de Matos, D. Modeling chronic pain experiences from online reports using the Reddit reports of chronic pain dataset. *Information* **14**, 237 (2023).

[24] Hallo-Carrasco, A. et al. Social media users’ perspectives of spinal cord stimulation: an analysis of data sourced from social media. *Reg Anesth. Pain Med* (2024). 10.1136/rapm-2024-105935 39455090

[25] Bian, J. et al. Mining Twitter to assess the public perception of the internet of things. *PLoS One*. **11**, e0158450 (2016). 27391760 10.1371/journal.pone.0158450 PMC4938510

[26] Ghosh, D. & Guha, R. What are we ‘tweeting’ about obesity? Mapping tweets with topic modeling and network analysis. *JMIR Public. Health Surveill*. **7**, e29078 (2021).

[27] Wu, J. et al. Trend and co-occurrence network of COVID-19 symptoms from large-scale social media data: infoveillance study. *J. Med. Internet Res.* **25**, e45419 (2023). 36812402 10.2196/45419 PMC10131634

[28] Sinnenberg, L. et al. Twitter as a tool for health research: a systematic review. *Am. J. Public. Health*. **107**, e1–e8 (2017). 27854532 10.2105/AJPH.2016.303512 PMC5308155

[29] Okui, N. Comorbid bladder pain syndrome and vulvodynia – a cross-sectional analysis of the UNICORN-4 study. *BMC Womens Health*. **25**, 72 (2025). 39972456 10.1186/s12905-025-03602-9 PMC11837444

[30] Okui, N. & Okui, M. A. The importance of psychological assessment in the management of bladder pain syndrome/interstitial cystitis and vulvodynia: a case report. *Cureus* **16**, e63617 (2024). 39092346 10.7759/cureus.63617 PMC11290954

[31] Okui, N., Okui, M. & Gambacciani, M. Examining vaginal and vulvar health and sexual dysfunction in patients with interstitial cystitis (UNICORN-1 study). *Int. Urogynecol. J.* **33**, 2493–2499 (2022). 35543734 10.1007/s00192-022-05220-7

[32] Okui, N. BPS/IC and vulvodynia: a comprehensive review of laser treatments and common pathophysiological pathways. *Curr. Bladder Dysfunct. Rep.* **19**, 330–339 (2024).

[33] Bosio, S. et al. The association between vulvodynia and interstitial cystitis/bladder pain syndrome: a systematic review. *Int. J. Gynecol. Obstet.* **167**, 1–15 (2024). 10.1002/ijgo.15538 38655714

[34] Peters, K. et al. Prevalence of pelvic floor dysfunction in patients with interstitial cystitis. *Urology* **70**, 16–18 (2007). 17656199 10.1016/j.urology.2007.02.067

[35] Yu, W. R. et al. Pelvic floor muscle pain is associated with higher symptom scores and bladder pain perception in women with interstitial cystitis and bladder pain syndrome. *World J. Urol.* **43**, 9–16 (2024). 39625572 10.1007/s00345-024-05366-7

[36] Gardella, B. et al. Interstitial cystitis is associated with vulvodynia and sexual dysfunction — a case-control study. *J. Sex. Med.* **8**, 1726–1734 (2011). 21477020 10.1111/j.1743-6109.2011.02251.x

[37] Fariello, J. Y. & Moldwin, R. M. Similarities between interstitial cystitis/bladder pain syndrome and vulvodynia: implications for patient management. *Transl Androl. Urol.* **4**, 643–652 (2015). 26816866 10.3978/j.issn.2223-4683.2015.10.09 PMC4708545

[38] Bornstein, J. et al. 2015 ISSVD, ISSWSH, and IPPS consensus terminology and classification of persistent vulvar pain and vulvodynia. *J. Low Genit. Tract. Dis.* **20**, 126–130 (2016). 27002677 10.1097/LGT.0000000000000190

[39] Offiah, I., McMahon, S. B. & O’Reilly, B. A. Interstitial cystitis/bladder pain syndrome: diagnosis and management. *Int. Urogynecol. J.* **24**, 1243–1256 (2013). 23430074 10.1007/s00192-013-2057-3

[40] Meister, M. R. et al. Development of a standardized, reproducible screening examination for assessment of pelvic floor myofascial pain. *Am. J. Obstet. Gynecol.* **220**, 255e1–255e9 (2019). 10.1016/j.ajog.2018.11.1106 PMC6401230 30527941

[41] Okui, N., Hachiya, T., Horie, S. & & Pilot study using a discrete mathematical approach for topological analysis and SsGSEA of gene expression in autosomal recessive polycystic kidney disease. *Sci. Rep.* **15**, 15559 (2025). 40319097 10.1038/s41598-025-99048-y PMC12049503

[42] Okui, N. Laser treatment for urinary incontinence in elite female athletes analyzed using a discrete mathematics approach. *Sci. Rep.* **15**, 15450 (2025). 40316601 10.1038/s41598-025-00363-1 PMC12048495

[43] Okui, N. & Okui, M. Discrete mathematics in dynamic network analysis: long-term efficacy evaluation of fotona laser therapy for overactive bladder syndrome using clustering-based patient subgroup identification. *Cureus* **16**, e68671 (2024). 39371818 10.7759/cureus.68671 PMC11452321

[44] Okui, N. Navigating treatment choices for stress and urgency urinary incontinence using graph theory in discrete mathematics. *Cureus* **16**, e61315 (2024). 38947730 10.7759/cureus.61315 PMC11213272

