# Supplementary material for: Discrete mathematical network analysis bridging clinical vocabulary and patient discourse in interstitial cystitis/bladder pain syndrome online communications
Source: Sci Rep. 2025 Nov 29;16:1251. doi: 10.1038/s41598-025-30819-3 (PMC12789557; doi:10.1038/s41598-025-30819-3)
Supplement: Supplementary file 1 — Supplementary Material 1 [file 41598_2025_30819_MOESM1_ESM.pdf]

Discrete mathematical network analysis bridging clinical vocabulary and patient discourse in interstitial cystitis/bladder pain syndrome online communications

Supplementary Information

Supplementary Table S1: Complete 19-term clinical vocabulary extracted from validated IC/BPS questionnaires

This table lists all symptom-related terms systematically extracted from three validated questionnaires (PUF, ICSI, ICPI), categorized into pain-related, urinary function, impact/bothering, and anatomical site terms. "Stage 1 Extracted" indicates inclusion in the reference vocabulary. "Stage 2 Frequency" shows total occurrences in the Reddit dataset (n = 525,465 words). "Detection Status" denotes whether the term was observed in patient discourse analysis. Undetected terms may reflect linguistic variation or low occurrence in patient discourse. Abbreviations: PUF = Pelvic Pain and Urgency/Frequency Patient Symptom Scale; ICSI = Interstitial Cystitis Symptom Index; ICPI = Interstitial Cystitis Problem Index. See corresponding Table 1 in the main manuscript for summary values.

Term	Category	Stage 1 Extracted	Stage 2 Frequency	Detection Status
pain	Pain	✓	2,034	Detected
bladder	Anatomical	✓	2,217	Detected
symptoms	Impact	✓	1,353	Detected
need	Urinary	✓	1,327	Detected

urinate	Urinary	✓	426	Detected
avoid	Impact	✓	281	Detected
burning	Pain	✓	71	Detected
vagina	Anatomical	✓	49	Detected
pelvis	Anatomical	✓	40	Detected
abdomen	Anatomical	✓	30	Detected
urethra	Anatomical	✓	24	Detected
urgency	Urinary	✓	7	Detected
discomfort	Pain	✓	3	Detected
pressure	Pain	✓	1	Detected
frequent	Urinary	✓	0	Not detected
bother	Impact	✓	0	Not detected
perineum	Anatomical	✓	0	Not detected
sacrum	Anatomical	✓	0	Not detected
testes	Anatomical	✓	0	Not detected

Supplementary Table S2: Strong co-occurrence pairs among validated vocabulary terms

This table shows term pairs meeting the strong-link criterion (direct co-occurrence, enhanced weight ≥90th percentile among all network edges) in Stage 3 analysis of Reddit IC/BPS discourse. "Enhanced Weight" includes clinical bonus scoring applied to validated term pairs. "Clinical Significance" summarizes the symptom–symptom or symptom–site association. Abbreviations: IC/BPS = interstitial cystitis/bladder pain syndrome. See corresponding Table 3 in the main manuscript for summary values.

Rank	Term U	Term V	Enhanced Weight	Percentile	Clinical Significance
1	pain	symptoms	177	97.2%	Core symptom reporting

2	pain	urinate	163	97.1%	Pain-voiding association
3	need	urinate	110	96.4%	Urgency-voiding cluster
4	pain	need	54	94.3%	Pain-urgency linkage
5	burning	urethra	50	93.9%	Anatomical localization
6	burning	urinate	37	92.0%	Burning-voiding pattern
7	burning	bladder	37	92.0%	Burning-bladder association
8	pain	abdomen	31	90.5%	Abdominal pain extension
9	burning	pain	30	90.2%	Pain type correlation

Supplementary Table S3: Validation of network metrics

This table compares key network analysis metrics reproduced from the analysis pipeline with values reported in the main manuscript, confirming reproducibility. Metrics include vocabulary detection rate, network density, clustering coefficient, community count, and centrality measures for "pain" and "bladder," as well as results of statistical comparisons between clinical and general vocabulary. See corresponding Tables 2 and text for related analysis.

Metric	Paper Value	Reproduced	Status
Vocabulary Detection Rate	73.7%	73.7%	✓
Network Density	0.0036	0.0036	✓

Clustering Coefficient	0.4108	0.4108	✓
Community Count	90	88-90	✓
pain Degree Centrality	0.2307	0.2307	✓
bladder Degree Centrality	0.2005	0.2005	✓
Clinical vs General t-test (degree)	t=17.30, p<0.001	t=17.30, p<0.001	✓
Clinical vs General t-test (betweenness)	t=11.31, p<0.001	t=11.31, p<0.001	✓
